# Excessive Formation and Stabilization of Dendritic Spine Clusters in the *MECP2*-Duplication Syndrome Mouse Model of Autism

**DOI:** 10.1523/ENEURO.0282-20.2020

**Published:** 2021-01-28

**Authors:** Ryan Thomas Ash, Jiyoung Park, Bernhard Suter, Huda Yaya Zoghbi, Stelios Manolis Smirnakis

**Affiliations:** 1 Department of Psychiatry and Behavioral Sciences, Stanford University, Stanford, CA 94305; 2 Department of Neuroscience, Baylor College of Medicine, Houston, TX 77030; 3 Medical Scientist Training Program, Baylor College of Medicine, Houston, TX 77030; 4 Department of Neurology, Brigham and Women’s Hospital, Harvard Medical School, Boston, MA 02115; 5 Department of Pediatrics, Texas Children’s Hospital and Baylor College of Medicine, Houston, TX 77030; 6 Department of Molecular and Human Genetics, Baylor College of Medicine, Houston, TX 77030; 7 Jan and Dan Duncan Neurological Research Institute at Texas Children’s Hospital, Houston, TX 77030; 8 Howard Hughes Medical Institute, Baylor College of Medicine, Houston, TX 77030

**Keywords:** autism, dendritic spine, MECP2, motor cortex, neuroplasticity, structural plasticity

## Abstract

Autism-associated genetic mutations may perturb the balance between stability and plasticity of synaptic connections in the brain. Here, we report an increase in the formation and stabilization of dendritic spines in the cerebral cortex of the mouse model of *MECP2*-duplication syndrome, a high-penetrance form of syndromic autism. Increased stabilization is mediated entirely by spines that form cooperatively in 10-μm clusters and is observable across multiple cortical areas both spontaneously and following motor training. Excessive stability of dendritic spine clusters could contribute to behavioral rigidity and other phenotypes in syndromic autism.

## Significance Statement

The inflexible repetitive behaviors, “insistence on sameness,” and at times exceptional learning abilities seen in autism imply a defect in the neural processes underlying learning and memory, potentially affecting the balance between stability and plasticity of synaptic connections in the brain. Here, we report a pathologic bias toward stability of newly formed dendritic spines in the *MECP2*-duplication mouse model of autism. Enhanced spine stability is mediated entirely by spines aggregating within 10 μm of each other, in clusters. Enhanced clustered spine stability is observable in multiple brain areas both at rest and during motor training. The results suggest that some phenotypes of autism could arise from abnormal consolidation of clustered synaptic connections.

## Introduction

It has been proposed that phenotypes of autism spectrum disorder arise from an abnormal imbalance between the stability and plasticity of synaptic connections in the brain (Ramocki and Zoghbi, 2008). Such an imbalance may potentially contribute to the rigid, restricted behavioral repertoire and insistence on sameness seen in autism (Howlin et al., 2009; Treffert, 2014). Rebalancing synaptic stability and plasticity could provide a therapeutic avenue to promote behavioral flexibility in patients.

Methyl-CpG-binding protein 2 (MeCP2) is an X-linked transcriptional regulator that contributes to the maintenance of neural circuit homeostasis through the activity-dependent modulation of gene expression and splicing (Lyst and Bird, 2015). Loss of function mutations in *MECP2* cause Rett Syndrome, a neurodevelopmental disorder affecting females (Chahrour and Zoghbi, 2007). Mice engineered to overexpress *MECP2* at twice normal levels exhibit a neurologic phenotype that falls squarely within the spectrum of autism (Collins et al., 2004; Samaco et al., 2012). They exhibit social avoidance, stereotypies, and behavioral inflexibility that progresses to motor dysfunction, spasticity, and epilepsy (Collins et al., 2004; Na et al., 2012; Samaco et al., 2012; Sztainberg et al., 2015). Patients with genomic duplication of *MECP2*, identified soon after the description of *MECP2*-duplication syndrome in mice, demonstrate many similar features highly characteristic of autism (Ramocki et al., 2009, 2010; Peters et al., 2013). The *MECP2*-duplication mouse is therefore a valuable tool for studying neural circuit phenotypes in autism (Tordjman et al., 2007).

An abnormal upregulation in the spontaneous turnover of pyramidal neuron dendritic spines has been observed across several mouse models of autism including the *MECP2*-duplication mouse (Chow et al., 2009; Cruz-Martín et al., 2010; Jiang et al., 2013; Isshiki et al., 2014; Gdalyahu et al., 2015; Ash et al., 2018), while mouse models of Rett syndrome exhibited decreased dendritic spine motility (Landi et al., 2011), suggestive of an abnormal balance between synaptic stability and plasticity. Interestingly, in the fragile X syndrome mouse model, it was found that new spines are less likely to be formed and stabilized in clusters compared with controls (Padmashri et al., 2013; Reiner and Dunaevsky, 2015), which could have interesting implications for abnormal learning and plasticity in autism (Yang et al., 2009; Fu et al., 2012). How other autism-associated mutations affect the spatial cooperativity of spine clusters, i.e., local plasticity-promoting interactions between spines mediated by molecular signaling cascades (Harvey and Svoboda, 2007; Harvey et al., 2008), has not been thoroughly evaluated.

In what follows, we provide evidence for an abnormal increase in the stabilization of cooperatively-formed neighboring dendritic spines in the *MECP2*-duplication syndrome mouse model. Excessive stabilization of spine clusters, but not of isolated spines, occurs in the motor and visual cortex of mutant animals, and is observable both in resting animals and animals repetitively trained on the rotarod motor task.

## Materials and Methods

### Animals

FVB-background *MECP2*-duplication (Tg1) mice (Collins et al., 2004), were crossed to C57 thy1-GFP-M homozygotes obtained from The Jackson Laboratory, to generate F1C57;FVB *MECP2*-duplication;thy1-GFP-M mice and thy1-GFP-M littermate controls. Males were used in experiments, to avoid possible changes in turnover related to estrous cycle in females (Alexander et al., 2018). Animals were housed in a 12/12 h light/dark cycle (lights on from 7 A.M. to 7 P.M.). All experiments with animals were conducted in accordance with the National Institutes of Health *Guidelines for the Care and Use of Laboratory Animals* and were approved by the Institutional Animal Care and Use Committee.

### Blinding

Surgeries, imaging, rotarod training, and analyses were performed blind to genotype. Mouse numbers and genotypes were placed in a two-column spreadsheet by a lab member not involved in the experiment or analysis, and MATLAB scripts imported genotypes from this spreadsheet to plot data without unblinding the experimenter to genotypes.

### *In vivo* two-photon imaging

Surgeries and imaging were performed blind to genotype. At least two weeks before the first imaging session (∼12- to 14-week-old mice), a 3-mm-wide opening was drilled over motor cortex, centered at 1.6 mm lateral to bregma based on Tennant et al. (2011), and a glass coverslip was placed over the exposed brain surface to allow chronic imaging of neuronal morphology (Mostany and Portera-Cailliau, 2008, 2011). Dendritic spines were imaged using a Zeiss *in vivo* two-photon microscope with Zeiss 20× 1.0 NA water-immersion objective lens. High-quality craniotomies had a characteristic bright-field appearance with well-defined vasculature and pale gray matter. Under two-photon scanning fluorescent dendrites were reliably clear and visible with low laser power (<20 mW on the pia) and photomultiplier tube voltage.

Only high-quality preparations (low background noise across all time points, less than five-pixel [0.25 μm] motion artifact, and dendrites well isolated from other fluorescent structures) were used in the blinded analysis (Movie 1). GFP-labeled neurons were first imaged at low resolution [620 × 620 μm field of view (FOV), 0.6 μm/pixel in *xy*, 2.5 μm *Z*-step size] down to 600–700 μm to capture all of the cell’s dendritic processes and assay cell subtype by morphology, primary apical bifurcation depth, and soma depth (Holtmaat et al., 2006). The apical dendrites from complex-tufted neurons, the corticospinal neurons projecting to the spinal cord and thalamus in M1, were selected based on their large highly ramified dendritic trees, deep primary apical bifurcation, and thick dendrites, and reimaged at high resolution (310 × 310–420 × 420 μm FOV, 0.1 μm/pixel, 1 μm *Z*-step size) to adequately capture individual dendritic spines. Laser power was maintained under 20 mW (average ∼10 mW) during image stack acquisition.

Movie 1. Example *z* stack of raw data acquired by *in vivo* structural imaging, demonstrating characteristic sparse, brightly fluorescent, L5 pyramidal neuron dendrites with clearly resolvable dendritic spines. 10.1523/ENEURO.0282-20.video.1

### Data inclusion criteria

Data inclusion was determined by the following indicators of imaging quality: (1) low background noise across all time points; (2) less than five-pixel (0.25 μm) motion artifact; and (3) dendrites well isolated from other fluorescent structures. Mice for which imaging quality significantly degraded during the time course of imaging were excluded from the analysis. Otherwise all data were included, and no statistical outliers were excluded.

### Analysis of structural plasticity

Raw *z* stacks were denoised by a custom polynomial interpolation method. Spine formation, elimination, and stabilization were quantified with a custom MATLAB user interface and ImageJ. Terminal dendrite segments which were well visualized at all time points were chosen for analysis. Sections of dendrite occluded by other fluorescent structures or blood vessels were excluded from the analysis. Because of *in vivo* two-photon microscopy’s relatively poor resolving power in the *z*-axis, only structures protruding laterally along the *x-y* plane were included in the analysis, following the standard in this field (Holtmaat et al., 2009). For a protrusion to be selected for analysis, it had to project out of the dendritic shaft by at least four pixels (∼0.4 μm), which corresponds approximately to 2 SDs of the noise blur on either side of the dendritic shaft. Spines were initially identified at one time point, by moving up and down through individual slices in each *z* stack, and the same region of dendrite was examined at other time points to identify the first (formation) and last (elimination) time that the spine was present. Custom MATLAB routines analyzed the stability/survival of each formed spine. Filopodia, which are rare at the analyzed age, were identified morphologically, based on their long length (usually >4 μm), curved shape, and lack of a distinct head (Zuo et al., 2005) and excluded from the analysis as in Yang et al. (2009).

### Analysis of dendritic spine clustering

Each spine was classified as either clustered or isolated by calculating the distance to its nearest-neighbor newly-formed stabilized spine (Fu et al., 2012). The difference in clustered spine stabilization rate between *MECP2*-duplication and wild-type (WT) mice was calculated as a function of cluster distance threshold (maximum distance to nearest co-stabilized spine to be counted as clustered; Fig. 3*A*). This difference in clustered spine stabilization increased with increasing cluster distance threshold and plateaued at 9 μm; we used this threshold to define spine clusters for further analysis.

### Spine clustering simulations

Simulations of formed, stabilized, and baseline dendritic spine locations were generated based on the measured mean and SD of dendritic length, the number of baseline spines and formed spines, and the spine stabilization rate in each genotype. Spine locations were simulated in one dimension. The minimum interspine distance of simulated spines was 0.1 μm (the minimum interspine distance observed in the data set); if a spine was <0.1 μm from another simulated spine, it was placed again until it met this criterion. Spine clustering was simulated per animal matched to the number of animals and number of dendrites imaged per animal in the experimental data set. Nearest neighbor analysis was performed on simulated dendritic spines to generate distributions of interspine distances per animal which were then averaged across animals as in Figure 2. The simulation was performed 1000 times to generate bootstrap expected values of interspine distances given random spine localization; 95% confidence intervals of the simulated results are plotted as maroon (mutant) and gray (WT) error bars in Figure 2.

### Motor training

Mice were imaged in the training and rest conditions in cohorts by litter. All animals within a litter were imaged in either the training or rest condition blind to genotype.

The Ugo Basile mouse rotarod was used for motor training. At least 2 h after imaging sessions, in the late afternoon, mice were placed on the rotarod, and the rotarod gradually accelerated from 5 to 80 rpm over 3 min. Single-trial rotarod performance was quantified as the time right before falling or holding on to the dowel rod for two complete rotations without regaining footing. A 5- to 10-min rest period occurred between each trial. Four trials were performed per day.

### Microstimulation mapping of motor cortex

**Figure 1.**
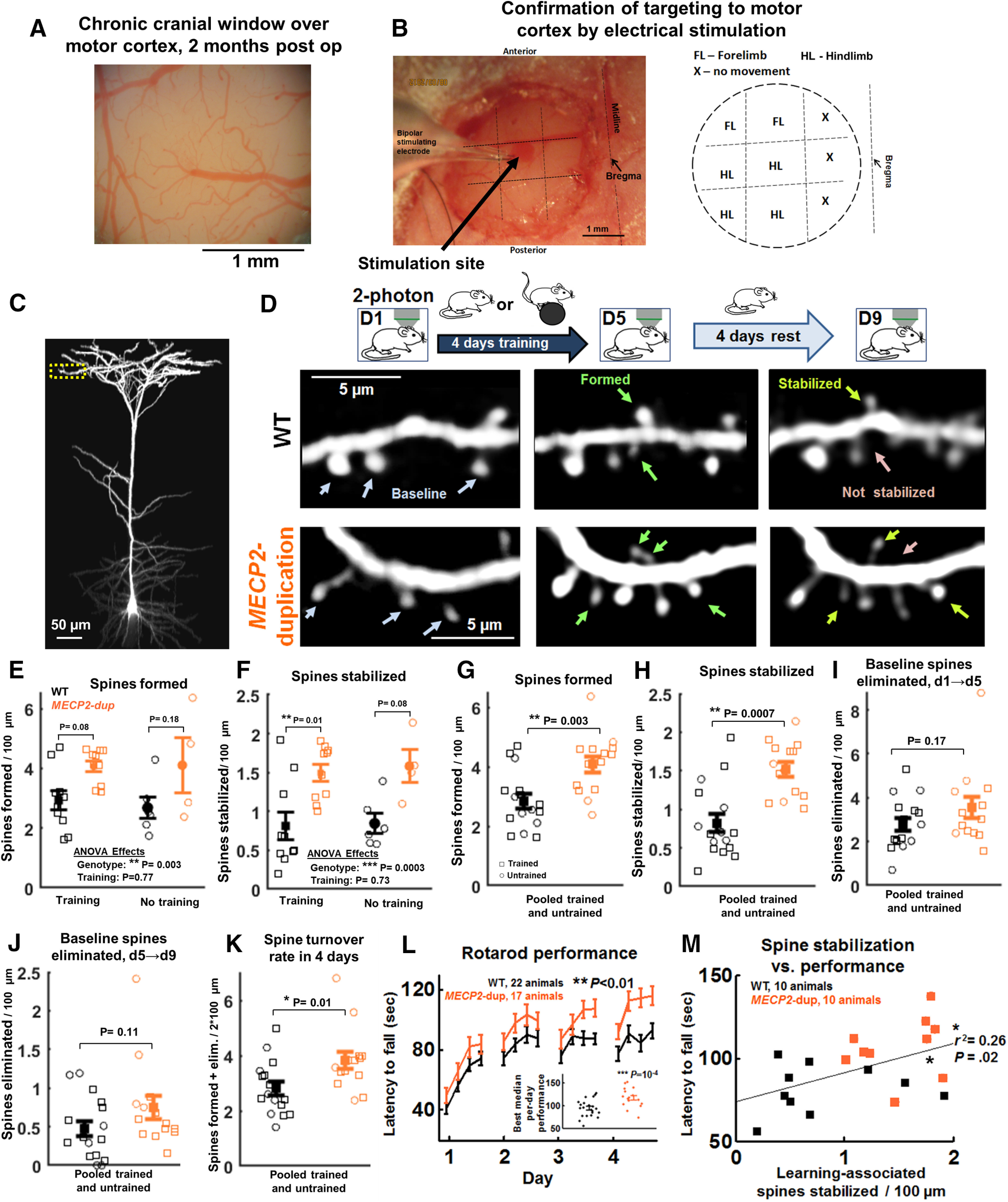
Increased formation and stabilization of dendritic spines in L5 pyramidal neuron apical dendrites of *MECP2*-duplication mice. ***A***, Example brightfield image of chronic cranial windows over M1 at two months postoperative, showing the well-defined vasculature and pale gray matter characteristic of high-quality preparations. ***B***, left, Image demonstrating the microstimulation experiment, performed *post hoc* in experimental mice at the end of imaging. A bipolar stimulating electrode was lowered ∼750 μm into the window at nine sites in a 1000-μm grid. Right, Example map of motor cortex generated by microstimulation. Forepaw and hindpaw twitches were generated at low currents in all stimulated cortices (*n* = 10 mice), confirming localization to M1. ***C***, The dendritic tree of a GFP-labeled L5 complex-tufted pyramidal neuron in area M1 imaged by *in vivo* two-photon microscopy. Apical tuft terminal dendrites are targeted for time-lapse imaging (yellow box). ***D***, Experiment paradigm: sample images of dendritic segments imaged at baseline (left), 4 d later (middle), and 8 d later (right). Some mice were trained on the rotarod daily while others were left to rest in the cage between D1 and D5. Top, WT controls. Bottom, *MECP2-*duplication animals. White arrows point to spines present at baseline, green to newly-formed spines, light-green to stabilized spines, and pink to non-stabilized spines. ***E***, Spines formed per 100 μm with and without motor training in terminal dendritic branches of *MECP2*-duplication animals (orange, training: *n* = 10 animals, 300 spines formed, 82 dendritic branches; no training: *n* = 4 animals, 56 spines formed, 27 dendritic branches) and WT littermate controls (black, training: *n* = 10 animals, 276 spines formed, 99 dendritic branches; no training: *n* = 6 animals, 42 spines formed, 35 dendritic branches). Squares and circles depict individual data points for trained and untrained animals, respectively. Effect of genotype: ** *p *=* *0.003, *F*_(1,26)_ = 10.3; effect of training: *p *=* *0.77; interaction: *p *=* *0.7; two-way ANOVA across animals); *p* values in plot show Tukey-corrected pairwise ANOVA comparisons. ***F***, Newly-formed spines stabilized per 100 μm in each genotype. Effect of genotype: ***p =* *0.0003, *F*_(1,26)_ = 17; training: *p *=* *0.7; interaction: *p *=* *0.88; *p* values in plot show Tukey-corrected pairwise ANOVA comparisons**. *G–K***, Dendritic spines formed, stabilized, and eliminated, and overall spine turnover rate in L5 apical dendritic arbors with data pooled between trained and untrained animals, in *MECP2*-duplication mice (orange: *n* = 14 animals, 356 spines formed, 109 dendritic branches) and WT littermate controls (black: *n* = 16 animals, 318 spines formed, 134 dendritic branches). Squares and circles depict individual data points for trained and untrained animals, respectively. ***G***, Spines formed per 100 μm, **p =* *0.003, Mann–Whitney *U* test. ***H***, Newly-formed spines stabilized per 100 μm, ***p =* *0.0007. ***I***, Baseline spines eliminated between experiment days 1 and 5, *p *=* *0.17. ***J***, baseline spines eliminated between experiment days 5 and 8, *p *=* *0.12. ***K***, Overall spine turnover rate (spines formed + spines eliminated/2 × total dendritic length) in 4 d (from day 1 to day 5), *p =* *0.01. ***L***, Mean per-trial rotarod performance (time spent on the accelerating rotarod before falling) across animals in *MECP2*-duplication animals (orange, *n* = 17) and WT controls (black, *n* = 22). This panel contains a larger sample size than other panels, because mice excluded from imaging analysis because of poor imaging quality but who still underwent rotarod training were included; **p =* *0.009, effect of genotype, *F*_(1,37)_ = 7.6, repeated-measures ANOVA. Effect of time: *p *<* *0.0001, *F*_(15,37)_ = 29.8; interaction: *p *=* *0.47, *F*_(15,37)_ = 0.99. ***M***, Scatter plot of learning associated spines that stabilized versus rotarod performance per animal, in imaged *MECP2*-duplication mice (orange squares) and WT controls (black squares); *p <* *0.05, *r*^2^ = 0.26, *n* = 20 mice pooled across genotypes, Pearson correlation, Student’s *t* test. Error bars indicate mean ± SEM. See Extended Data Figure 1-1 for visualization of data as estimation plots with bootstrap-estimated differences between groups (see Materials and Methods).

After the final imaging session, microstimulation mapping of motor cortex was performed (Fig. 1*B*) as described previously (Tennant et al., 2011). Under ketamine/xylazine anesthesia, the coverslip was removed from the craniotomy, and a bipolar stimulating electrode was lowered into the imaged brain region in a 0.5 × 0.5-mm grid pattern to ∼750-μm depth (deep L5). Current pulses starting at 10 μA (up to 60 μA) were applied until twitching was observed in contralateral muscle groups. Twitching muscle groups were scored as forelimb, hindlimb, face, tongue, or tail. Imaged dendritic arbors were registered with mapped regions using blood vessels as landmarks. All structural plasticity data were acquired from forelimb and hindlimb motor cortex.

10.1523/ENEURO.0282-20.2020.f1-1 Extended Data Figure 1-1 Dendritic spine structural plasticity estimation statistics. ***A–E***, Same as Figure 1*G–K*, but plotted as a Gardner–Altman estimation plot to visualize the results using estimation statistics as in Ho et al. (2019). The left axis of each panel shows individual data points for WT (black) and *MECP2*-duplication (orange) animals. The right axis shows the bootstrapped distribution (light orange) and 95% confidence interval (vertical black line) of the estimated difference between the two groups; *p < 0.05, **p < 0.01, ***p < 0.001, Mann–Whitney *U* test. Download Figure 1-1, TIF file.

### Image presentation

Dendritic spine images are displayed as “best” projection mosaics. Extraneous fluorescence is masked and images are slightly smoothed for illustration purposes only.

### Data visualization

To more rigorously illustrate variability in the data and statistical robustness, data were plotted as Gardner–Altman and Cummings estimation plots, as previously shown (Bernard, 2019; Calin-Jageman and Cumming, 2019; Ho et al., 2019). Raw data were exported to https://www.estimationstats.com/, and graphs were generated. Bootstrap sampling distributions were generated with five thousand bootstrap samples; bootstrap confidence intervals were bias-corrected and accelerated.

### Statistical tests

Statistical significance between samples were assessed by two-way ANOVA or Mann–Whitney *U* test, as noted, using MATLAB. All results are reported as mean ± SEM, unless otherwise noted.

## Results

### Increased dendritic spine stabilization in *MECP2*-duplication mice

We employed *in vivo* two-photon microscopy (Holtmaat et al., 2009) in primary motor cortex (M1) to measure changes in dendritic spine structural plasticity in the *MECP2*-duplication mouse *in vivo*. Chronic cranial windows were implanted over M1 in *MECP2*-duplication mice and littermate controls (Fig. 1*A*,*B*). Apical dendrites from GFP-expressing (Feng et al., 2000) L5 pyramidal neurons in area M1 (Grimsley et al., 2013) were targeted for imaging (Fig. 1*C*; Movie 1; Holtmaat et al., 2009). Spine analysis was performed on terminal dendritic branches of the apical tuft of these neurons. Imaging and analysis were performed blind to genotype (see Materials and Methods). Correct targeting to area M1 was confirmed by electrical microstimulation after the final imaging session (Fig. 1*B*; Tennant et al., 2011). We first identified baseline spines, then animals either rested in their cage or were trained on the rotarod task (four trials per day) for 4 d (Fig. 1*D*). On the fifth day, we imaged the dendrites again to identify new spines formed. Following four more days of rest (the time frame of spine stabilization), dendrites were once again imaged to identify the new spines that stabilized. The follow-up imaging time point was chosen in line with prior studies showing that the vast majority of newly formed dendritic spines, which persist for at least 4 d, form an electron-microscopy-verified synapse (Knott et al., 2006).

We found that ∼30% more spines were formed in *MECP2*-duplication mice (Fig. 1*E*, orange) WT littermates (black; *p *=* *0.002, *F*_(1,26)_ = 11.3, effect of genotype, two-way ANOVA; Fig. 1*E*). Similar numbers of spines were formed in trained versus untrained animals in both genotypes (*p *=* *0.8, *F*_(1,26)_ = 0.04, effect of training; Fig. 1*E*), presumably because of the weak training paradigm employed relative to (Yang et al., 2009; Liston et al., 2013; Frank et al., 2018), which was selected to optimize the behavioral difference in learning between mutant and control mice (Collins et al., 2004). Almost twice as many new spines were stabilized in *MECP2*-duplication mice compared with controls (1.5 ± 0.1 vs 0.8 ± 0.2 spines/100 μm*, p *=* *0.0002, *F*_(1,26)_ = 17.9, effect of genotype, two-way ANOVA; Fig. 1*F*), an effect that was observable in both trained and untrained animals and also did not vary significantly with training (effect of training: *p *=* *0.7). Given the lack of effect of rotarod training on spine formation and stabilization for the training paradigm we selected, data from trained and untrained animals were pooled for the remainder of this report. Analyzed dendritic lengths were similar between genotypes (*p *=* *0.9, WT: 78 ± 36 μm, *MECP2*-duplication: 80 ± 34 μm, median ± SD).

With data pooled, we confirmed that significantly more spines were formed and stabilized in *MECP2*-duplication mice versus WT [formation: 4.1 ± 0.2 vs 2.8 ± 0.3 spines/100 μm*, p *=* *0.003, Mann–Whitney *U* test, *n* = 16 WT animals, *n* = 14 *MECP2*-duplication animals (Fig. 1*G*); stabilization: 1.5 ± 0.1 vs 0.8 ± 0.1 spines/100 μm, *p *=* *0.0007 (Fig. 1*H*)]. With data pooled, the study was well powered to detect significant differences in formation (1−β = 0.994) and stabilization (1−β = 0.9991) in *MECP2*-duplication mice. Data are visualized as estimation plots with bootstrap-estimated differences between groups as in Ho et al. (2019) in Extended Data Figure 1-1 (see Materials and Methods). These results indicate a pronounced increase in the formation and stabilization of dendritic spines in mutant animals.

There was a nonsignificant trend toward increased baseline spine elimination rate in *MECP2*-duplication mice versus littermate controls across the 8 d of imaging (Fig. 1*I*,*J*), although our study was not statistically powered to detect this difference given variability in WT animals (1−β = 0.60 for d1→5; 1−β = 0.64 for d5→9). Overall, spine turnover rates (spines formed + spines eliminated in 4 d per 2*100 μm) were significantly increased in mutants versus WT (*p *=* *0.01, Mann–Whitney *U* test, 1−β = 0.9445; Fig. 1*K*). For estimation plot visualizations of these data, see also Extended Data Figure 1-1*C–E*. These results cohere with our previous findings of increased spine turnover rate and spine elimination in the somatosensory cortex of *MECP2*-duplication mice (Jiang et al., 2013). Spine densities were similar between the three- to four-month-old *MECP2*-duplication mice and WT controls we imaged (WT: 0.2 ± 0.01 spines per micrometer; *MECP2*-duplication: 0.23 ± 0.1 spines per micrometer), also in agreement with results from somatosensory cortex at that age (Jiang et al., 2013). Previous longitudinal imaging from somatosensory cortex in *MECP2*-duplication mice found that spine density decreases gradually with age in these animals (Jiang et al., 2013), suggesting that the increase in stabilization of newly formed spines we report here (Fig. 1*G*) is not sufficient to compensate for the overall increase in spine turnover rate (Fig. 1*I–K*), leading to a net decrease in spine density over time.

*MECP2*-duplication mice performed significantly better on the rotarod as has been previously reported (Collins et al., 2004; *p *=* *0.01; WT: *n* = 22 animals, *MECP2*-duplication: *n* = 17; repeated-measures ANOVA; Fig. 1*K*). The median best per-day performance was 117 ± 5 s for *MECP2*-duplication mice and 94 ± 4 s for controls (*p *=* *10^−4^, *t* test across animals). Spine formation, stabilization, and elimination did not correlate strongly with motor performance in trained animals within either genotype (all *p *>* *0.5, linear regression), likely because of the fact that our training paradigm was weak (Yang et al., 2009). However, when data were pooled across genotypes, there was a significant correlation between spine stabilization and rotarod performance (*r*^2^ = 0.26, *p *=* *0.02, Pearson correlation, Student’s *t* test; Fig. 1*M*). Given that prior studies (Yang et al., 2009; Liston et al., 2013) have clearly shown that higher spine stabilization rate correlates with enhanced behavioral performance with rotarod training, this suggests that the increased rate of spine stabilization observed in *MECP2*-duplication animals may in part explain the higher rotarod performance of mutants versus controls.

### Increased stabilization of dendritic spine clusters in *MECP2*-duplication mice

**Figure 2.**
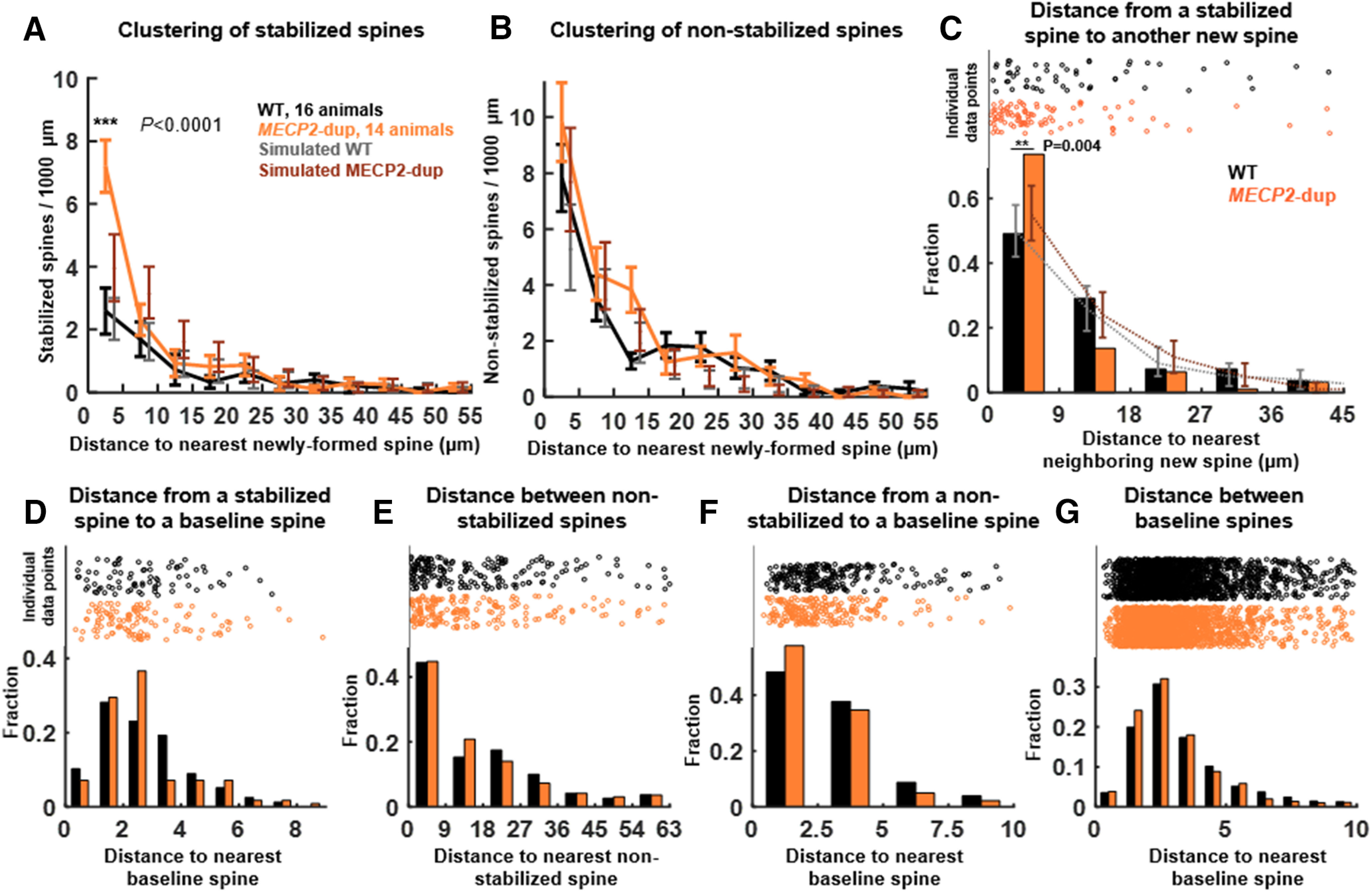
Increased stabilization of dendritic spine clusters in *MECP2*-duplication mice. ***A***, Mean number of new spines that stabilized per 1000 μm, binned by distance to the nearest neighboring new spine. Bin size = 5 μm; ***p <* *10^−7^, two-way ANOVA with Tukey correction for multiple comparisons, *F*_genotype(1,29)_ = 34.3, *F*_clusterbin(12,29)_ = 14.7, *F*_genotype × clusterbin(12,29)_ = 7.6, *n* = 16 WT, 14 *MECP2*-duplication animals. Gray and maroon error bars show mean ± 95% confidence intervals of spine distributions predicted by simulation based on measured spine formation and stabilization rates. ***B***, Mean number of spines that formed but did not stabilize per 1000 μm, binned by distance to the nearest neighboring new spine. Gray and maroon error bars depict simulation results. ***C***, Histogram of the distance to the nearest neighboring newly formed spine (whether stabilized or non-stabilized) for each stabilized spine, in *MECP2*-duplication (orange) and WT (black) mice. Individual data points for each spine are shown at the top; *p =* *0.004, Fisher exact test. Gray and maroon error bars depict mean ±95% confidence intervals of the estimated distribution of distances between stabilized spines simulated from the number of spines formed and stabilized per micrometer in each genotype (see Materials and Methods). Differences in overall spine formation and stabilization in mutants do not explain the increase in clustered spine stabilization between mutant and WT (*p *<* *0.001, *MECP2*-duplication data vs *MECP2*-duplication simulation, boot strap comparison). ***D***, Histogram of distances from each stabilized spine to the nearest preexisting baseline spine. ***E***, Histogram of nearest-neighbor distances between all non-stabilized new spines. ***F***, Histogram of distances from each non-stabilized spine to the nearest preexisting baseline spine. ***G***, Histogram of nearest-neighbor distances between baseline spines. None of the distributions in ***D–G*** showed significant differences. Error bars indicate mean ± SEM.

We next examined the spatial distribution of dendritic spines forming in motor cortex (Morita, 2009; Fu et al., 2012; Kastellakis et al., 2015; Frank et al., 2018). We observed that in *MECP2*-duplication mice newly formed spines were often stabilized in pairs or triplets along the dendrite (Fig. 1*D*, bottom right panel). We binned stabilized spines by their proximity to other newly formed spines and detected a dramatic increase in synaptic clustering in *MECP2-*duplication mice that was not present in WT littermates (Fig. 2*A*). Almost three times as many spines were stabilized within 5 μm of another new spine in *MECP2-*duplication mice compared with WT controls (7.2 ± 0.8 vs 2.6 ± 0.7 spines/1000 μm, *p *<* *0.0001, *n* = 14 *MECP2*-duplication, 16 WT animals, ANOVA with Tukey correction for multiple comparisons). Beyond 10-μm interspine distance, similar numbers of spines were stabilized in both genotypes, indicating that the increased spine stabilization observed in *MECP2*-duplication mice (Fig. 1*F*,*G*) is mediated almost exclusively through excessive stabilization of dendritic spine clusters (Fig. 2*A*).

Dendritic spine locations were simulated matched to measured spine formation and stabilization rates in mutants and controls and the number of analyzed dendrites and animals, to generate bootstrap confidence intervals of expected spine distributions given no spatial inhomogeneity in formation/stabilization (95% confidence intervals; Fig. 2*A*,*B*, gray and maroon error bars; for details, see Materials and Methods). Comparing simulated spine distributions to experimental data showed that increased clustered spine stability was not a by-product of the overall increase in spine formation or stabilization observed in mutants. Non-stabilized spines occurred in clusters at similar rates between WT mice and *MECP2*-duplication mice (Fig. 2*B*).

Plotting the distribution of interspine distances between each stabilized spine and its nearest neighbor newly formed spine confirmed an upregulation in the number of clustered stabilized spines in mutants compared with WT controls (*p *=* *0.004, Fisher exact test; Fig. 2*C*). The distance from each stabilized spine to the nearest baseline spine (Fig. 2*D*) did not differ significantly between *MECP2*-duplication mice and controls (*p* > 0.05, Fisher exact test). Newly formed non-stabilized spines (Fig. 2*E*,*F*) and baseline spines (Fig. 2*G*) also demonstrated spatial distributions similar to WT.

New spines were not more likely to be stabilized in clusters than chance in WT mice (Fig. 2*A–C*, compare data black lines to simulation gray lines), indicating that cooperative spine stabilization was specific to mutants under our experimental conditions. Separately analyzing data from trained and untrained animals did not show a significant effect of training on spine clustering in mutants or controls (effect of training: *p *=* *0.6, *F* = 0.29; genotype × training interaction: *p *=* *0.9, *F* = 0.01; two-way ANOVA), which we attribute either to the type or weak intensity of training employed (Fu et al., 2012).

**Figure 3.**
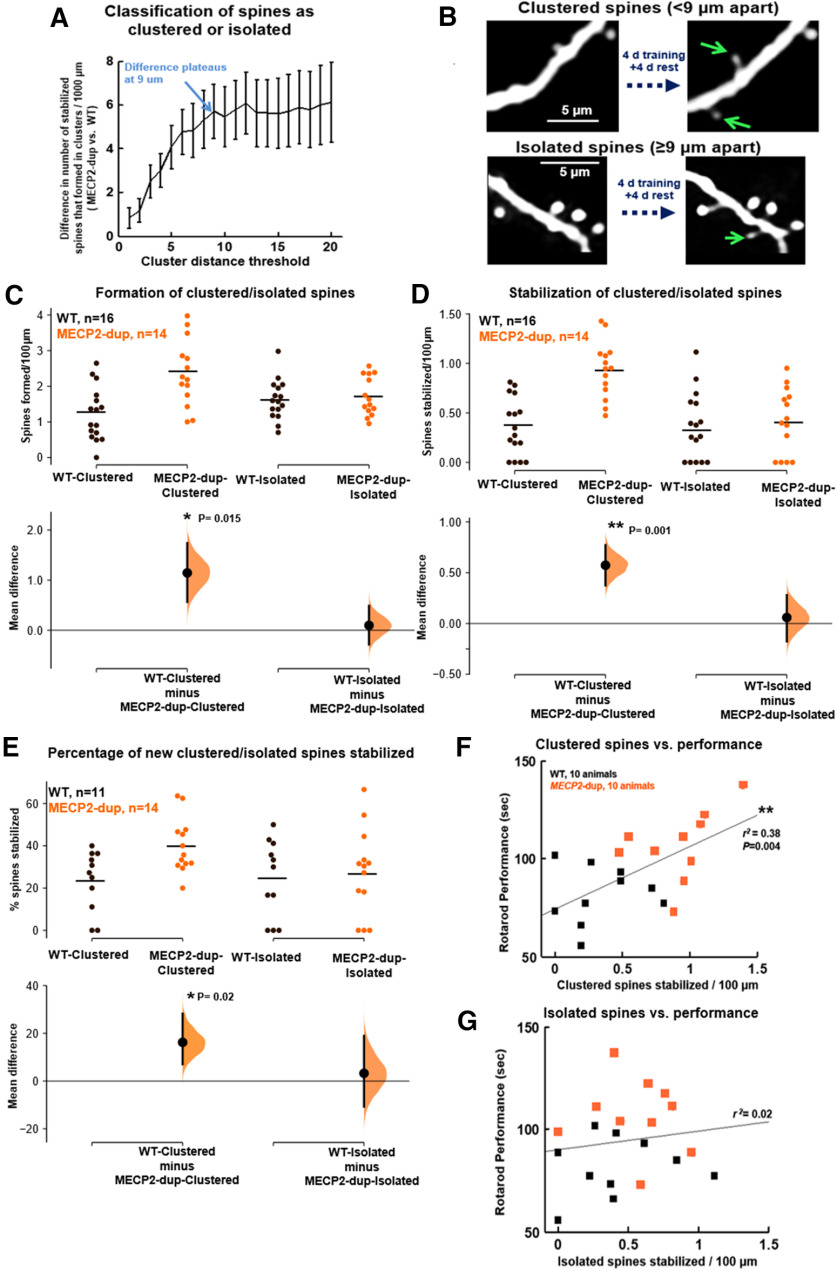
Differential stabilization of clustered and isolated spines in *MECP2*-duplication mice. ***A***, Determination of cluster distance threshold. Increase in clustered spine stabilization in mutants versus WT littermates, plotted as a function of the cluster distance threshold applied (i.e., the maximum distance to another newly formed spine to be categorized as clustered). Error bars represent the summed SEM from both genotypes. The difference in clustered spine stabilization between genotypes increases with increasing cluster distance threshold leveling off to a plateau at ∼9 μm; 9 μm was therefore chosen for further analysis, and this agrees with the range of spine consolidation cooperativity shown *in vitro* by Harvey and Svoboda (2007) and *in vivo* by Fu et al. (2012). ***B***, top, Example of stabilized clustered spines. Bottom, Example of an isolated stabilized spine. ***C–E***, Clustered and isolated spines formed per 100 μm, stabilized per 100 μm, and percentage stabilized from *MECP2*-duplication mice (orange) and WT (black), visualized as a Cumming estimation plot, as in Ho et al. (2019). Raw data are plotted on the upper axes (dots depict individual data points, horizontal lines depict means). The lower axes show the bootstrapped distribution (light orange) and 95% confidence interval (vertical error bar) of the estimated mean difference between the two groups. Data were pooled across trained and untrained animals. ***C***, Clustered and isolated spines formed per micrometer in each genotype; *p =* *0.015, Mann–Whitney *U* test. ***D***, clustered and isolated spines stabilized per micrometer in each genotype; **p =* *0.001. ***E***, Percentage of newly formed clustered and isolated spines that stabilized. Animals that formed fewer than four clustered spines were excluded from the analysis because the percentage measure shows large variability with small numbers of spines; *n* = 11 WT, *n* = 14 *MECP2*-duplication mice; *p =* *0.02. ***F***, Stabilization of dendritic spine clusters correlates with enhanced rotarod performance in *MECP2*-duplication mice (orange) and WT controls (black); **p =* *0.004, *r*^2^ = 0.38, *n* = 20 mice pooled across genotypes, Pearson correlation, Student’s *t* test**. *G***, Stabilization of isolated new spines does not correlate with rotarod performance (*r*^2^ = 0.02, *p *=* *0.55), suggesting that clustered spine stabilization is a better predictor of behavioral performance. For visualization of data as mean ± SE with individual data points plotted as circles (untrained) and squares (trained), see Extended Data Figure 3-1.

To further quantify clustered-spine stabilization in *MECP2*-duplication mice, we categorized each stabilized spine as clustered (<9 μm to nearest neighboring new spine; Fig. 3*A*,*B*) or isolated (≥9 μm to nearest neighboring new spine). Nine micrometers was chosen as a distance threshold for defining clusters because the difference in clustered-spine stabilization between mutants and WT plateaued at this threshold (Fig. 3*A*; see Materials and Methods); this distance also matches a known form of cooperative synaptic clustering observed *in vitro* (Harvey and Svoboda, 2007). This analysis further confirmed the upregulation in clustered but not isolated spine formation (*p *=* *0.01; Fig. 3*C*) and stabilization (*p *=* *0.001; Fig. 3*D*) in *MECP2*-duplication mice compared with controls. Similar to what was observed for overall spine formation and stabilization, analyzing trained and untrained animals separately showed no effect of training on clustered spine formation and stabilization (formation: ANOVA effect of training: *p *=* *0.72, effect of genotype: *p *=* *0.0008, interaction: *p* = 0.3; stabilization: ANOVA effect of training: *p = *0.59, effect of genotype: *p *<* *0.00,001, interaction: *p* = 0.9). Interestingly, clustered new spines were almost twice as likely to be stabilized in *MECP2*-duplication mice (40 ± 4% of clustered spines; Fig. 3*E*) compared with WT (23 ± 4% of clustered spines, *p *=* *0.02, Mann–Whitney *U* test), while isolated spines showed similar rates of consolidation between genotypes (Fig. 3*E*). Importantly, similar statistically significant results were observable for a range of cluster thresholds (from 5 to 12 μm). Mean ± SE error bars with individual data points plotted as circles (untrained) and squares (trained) are shown for these data in Extended Data Figure 3-1 for completeness.

10.1523/ENEURO.0282-20.2020.f3-1 Extended Data Figure 3-1 Clustered and isolated spine stabilization supplemental plots. ***A–C***, Same as Figure 3*C–E*, but plotted as mean ± error bars, with individual animal data points plotted as squares (trained) and circles (untrained). Download Figure 3-1, TIF file.

Previous work has shown that mice with elevated clustered dendritic spine plasticity are superior learners (Frank et al., 2018). Indeed, when pooling animals from both genotypes, we found that animals that formed and stabilized larger numbers of new spine clusters (typically *MECP2*-duplication mice) performed better on the rotarod (*r*^2^ = 0.38, *p *=* *0.004; Student’s *t* distribution; Fig. 3*F*). In contrast, stabilization of isolated new spines did not correlate with enhanced performance (*r*^2^ = 0.02, *p *=* *0.55; Fig. 3*G*). These results suggest that abnormal clustered-spine stabilization could contribute to enhanced procedural memory consolidation in *MECP2*-duplication mice.

**Figure 4.**
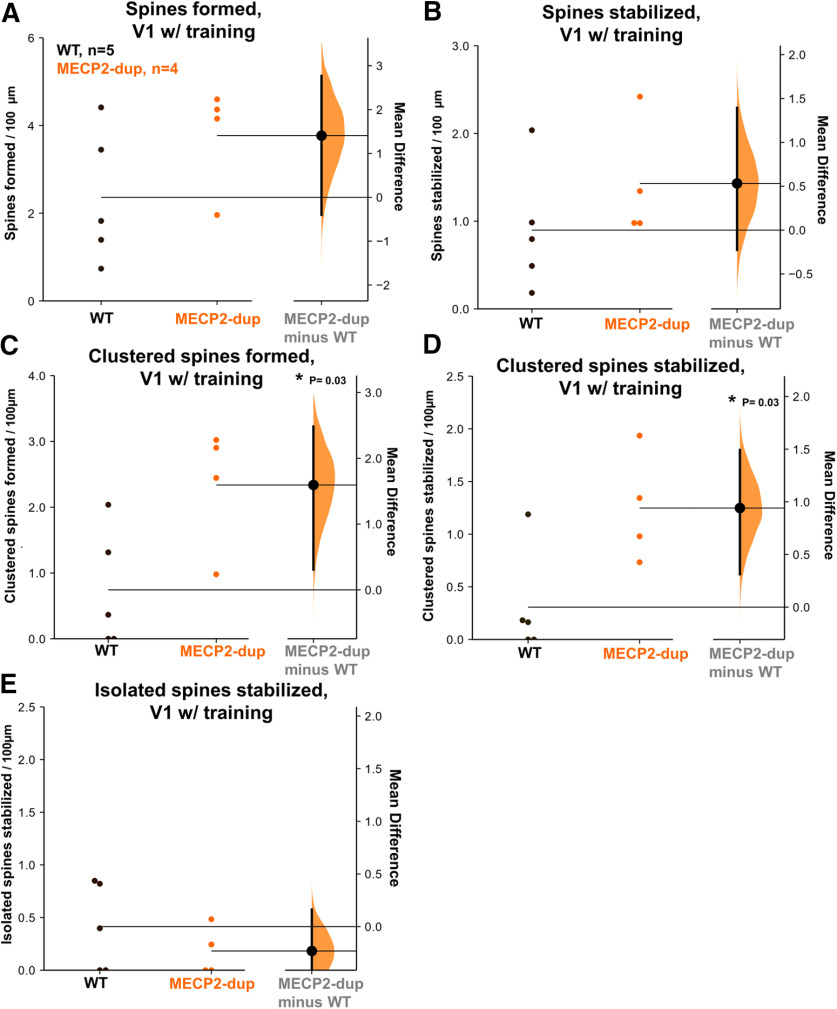
Dendritic spine structural plasticity in visual cortex (V1) in *MECP2*-duplication mice and littermate controls. ***A–E***, Spine formation and stabilization in visual cortex. Orange, *MECP2*-duplication. Data are plotted as a Gardner–Altman estimation plot to visualize the results using estimation statistics as in Ho et al. (2019). The left axis of each panel shows individual data points for WT (black) and *MECP2*-duplication (orange) animals. The right axis shows the bootstrapped distribution (light orange) and 95% confidence interval (vertical black line) of the estimated difference between the two groups. WT: *n* = 5 mice, 60 spines formed, 33 branches; *MECP2*-duplication: *n* = 4 mice, 60 spines formed, 31 branches. All animals in this condition were trained on the rotarod. ***A***, New spines formed between D1 and D5 per 100 μm in visual cortex in each genotype. ***B***, New spines stabilized (still present at D9) per 100 μm in visual cortex in each genotype. ***C***, Clustered spines formed per 100 μm in visual cortex in each genotype; *p =* *0.03, Mann–Whitney *U* test. ***D***, Clustered spines stabilized per 100 μm in visual cortex in each genotype; *p =* *0.03. ***E***, Isolated spines stabilized per 100 μm in each genotype. Error bars indicate mean ± SEM.

We next checked to see whether changes in clustered spine stabilization were observable in other cortical areas. We therefore performed the same imaging experiment in the visual cortex of mutants and controls (Fig. 4). Remarkably, although overall spine formation and stabilization were not significantly increased in *MECP2*-duplication mouse visual cortex (Fig. 4*A*,*B*), clustered spine formation (Fig. 4*C*) and stabilization (Fig. 4*D*) were also significantly upregulated in this area in mutants. As observed in motor cortex, isolated spine stabilization was similar between mutants and controls in primary visual cortex (V1; Fig. 4*E*). These data indicate that excessive clustered spine stabilization is driving spine consolidation in multiple cortical areas in *MECP2*-duplication mice.

## Discussion

Abnormally increased dendritic spine turnover has been observed in several autism mouse models (Chow et al., 2009; Jiang et al., 2013; Isshiki et al., 2014; Gdalyahu et al., 2015), including the *MECP2*-duplication, neuroligin-3, 15q duplication, PTEN, and CNTNAP2 mice, suggesting they share a deficit in the balance between structural synaptic plasticity and stability.

We found that ∼33% more new spines are formed after 4 d in apical tufts of *MECP2*-duplication mouse L5 pyramidal neurons compared with littermate controls. These newly-formed spines are ∼40% more likely to be stabilized compared with controls, leading to almost twice as many new spines stabilized in *MECP2*-duplication animals. Remarkably, the increased dendritic spine stabilization we observed in *MECP2*-duplication mice was mediated entirely by spines formed in 9-μm-long clusters. This clustering was specific to newly formed spines and was not observable in the spatial distribution of non-stabilized spines or baseline spines, nor was it observable in the distance between new spines and baseline spines, suggesting that cooperativity is specific to newly formed spines.

### Elevated clustered spine stabilization and increased spine turnover: implications for synaptic homeostasis

Although newly formed dendritic spine clusters were more stable in *MECP2*-duplication mice (Fig. 3*D*), overall dendritic spine turnover was elevated (Figure 1*K*; and spine elimination also trended toward higher values in mutants; Fig. 1*I*,*J*). These latter results agree with our prior studies in the somatosensory cortex of *MECP2*-duplication mice showing increased dendritic spine turnover and a net increase in spine elimination, with higher spine densities in young mice falling gradually with age to plateau at a lower spine density compared with control in older animals (>16 weeks). Therefore, there are two plasticity processes operating differently in *MECP2*-duplication mice: increased stabilization of clustered spines and increased overall spine turnover, which presumably serve to somewhat balance each other. It is interesting to speculate that one of these processes may reflect the primary defect, while the other could serve as a compensatory mechanism. Elevated spine turnover may explain why we do not observe an overall increase in clustering of “baseline spines,” i.e., spines detected on the first day of observation (Fig. 2*G*). Follow-up experiments will be important to assess dendritic spine consolidation over longer time scales in the future.

### Impact of increased synaptic clustering

An increase in clustered spine stabilization can potentially have important functional implications (Kastellakis et al., 2015; Gökçe et al., 2016; Weber et al., 2016). Clusters of synapses drive neuronal activity more strongly when activated synchronously through nonlinear dendritic integration mechanisms (Major et al., 2013). Neurons that implement synaptic clustering may fire selectively to precise combinations of inputs spatially co-localized on the dendrite, in theory dramatically increasing memory storage capacity (Poirazi and Mel, 2001). Too much input clustering, however, may potentially lead to “overfitting” of learned representations leading to a rigid and restricted behavioral repertoire that may not be flexible enough to accommodate the efficient learning of new representations (Collins et al., 2004; Na et al., 2012; Cai et al., 2020; Yu et al., 2020). Changes in input clustering could also contribute to changes in neuronal network correlation structure, excitability, and sensory processing (Lu et al., 2016; Nageshappa et al., 2016; Zhang et al., 2017; Zhou et al., 2019; Sun et al., 2020). We note that the above discussion depends on functional as well as cytological clustering of synaptic inputs, while at this point, we only show differences in cytological clustering of newly formed spines in *MECP2*-dupication mice.

### Presynaptic inputs

It is interesting to speculate on the origins of the presynaptic inputs to the newly-formed corticospinal apical-tuft dendritic spines we studied. Corticospinal neurons integrate information from premotor cortex, somatosensory cortex, and corticostriatal and corticocerebellar circuits to implement adaptive motor control (Kuramoto et al., 2009; Mao et al., 2011; Hooks et al., 2013). Previous work showed increased stability of presynaptic axonal boutons in L5 pyramidal neuron projections to layer 1 of motor cortex in mutant mice (Ash et al., 2018). This raises the possibility that these boutons could form synapses with newly formed spines, increasing the stability of the bouton-spine complex in *MECP2*-duplication mice. An interesting nonexclusive possibility is that newly formed synaptic clusters reflect multiple synaptic connections from a single presynaptic neuron (Kasthuri et al., 2015; Yang et al., 2016).

### Candidate mechanism of increased clustering

The molecular mechanisms driving increased clustered spine stabilization in mutants is a fascinating research question. Of particular interest is the Ras-MAPK pathway, which has been shown *in vitro* to be specifically involved in the cooperative potentiation of neighboring dendritic spines (Harvey et al., 2008; Patterson et al., 2010; Kwon and Sabatini, 2011). Ras-MAPK genes are dysregulated in *MECP2*-duplication mice (Chahrour et al., 2008), mutations in Ras-MAPK pathway genes are linked to several forms of autism (Stornetta and Zhu, 2011), and several autism models have been shown to have abnormal Ras-MAPK signaling (Ebert and Greenberg, 2013).

### Effects of training

We note that training on the rotarod did not lead to increased spine formation, stabilization or clustering in either genotype, nor did the number of spines stabilized during learning correlate strongly with motor performance separately for each genotype, in contrast to prior studies (Yang et al., 2009; Fu et al., 2012; Liston et al., 2013; Clark et al., 2018; Frank et al., 2018). We believe that the absence of a strong link between spine stabilization/clustering and motor learning within genotype is because of the fact that we employed a weak rotarod training paradigm of four trials per day for 4 d (vs 20 trials per day for 2 d as in Yang et al., 2009). Our paradigm was designed to maximize the behavioral difference between mutants and controls (as in Collins et al., 2004 and Sztainberg et al., 2015), but was not as effective in eliciting changes in spine formation or stabilization above the baseline observed without training. It is likely that repeated “overtraining” in a task is necessary to induce measurable increases in spine formation in area M1. Future work with stronger or different training paradigms (e.g., the seed-grabbing task in Fu et al., 2012) will allow a better controlled assessment of the relationship between enhanced clustered spine stabilization and enhanced motor learning in *MECP2*-duplication mice (Collins et al., 2004).

Because of its relative weakness, our paradigm did not elicit significant correlations between spine formation/stabilization and motor performance within each genotype. It did demonstrate a strong correlation between the propensity for spine stabilization (measured regardless of training) and motor performance, when genotypes were pooled together (Figs. 1*I*, 3*F*). Interestingly, this correlation was mediated entirely by spines that formed in clusters and not by the formation of new isolated spines (Fig. 3*F*,*G*). This observation suggests a link between the capacity to form spine clusters and behavioral performance (Frank et al., 2018). However, it remains an open question whether enhanced motor performance in *MECP2*-duplication animals is in fact causally because of their increased capacity for synaptic clustering.

Our findings illustrate how neural circuit analysis can generate new hypotheses about the pathophysiology of neurodevelopmental disorders. We demonstrated a marked increase in clustering of newly formed spines in *MECP2*-duplication animals, which occurs regardless of training and appears to be associated with the enhanced capacity for motor learning observed early-on in these animals. It remains an open question whether increased clustered-spine stability contributes causally to the motor phenotype of the mouse model of *MECP2*-duplication syndrome. It is also not clear whether/how the observed synaptic stability phenotype may contribute to some aspects of the human disorder. In the future, it will be valuable to explore further the proposition that a pathologic imbalance between synaptic stability and plasticity in different circuits might account for different phenotypic aspects of the *MECP2*-duplication syndrome and other autism spectrum disorders.

## Synthesis

Reviewing Editor: Christian Hansel, University of Chicago Decisions are customarily a result of the Reviewing Editor and the peer reviewers coming together and discussing their recommendations until a consensus is reached. When revisions are invited, a fact-based synthesis statement explaining their decision and outlining what is needed to prepare a revision will be listed below. The following reviewer(s) agreed to reveal their identity: Ricardo Mostany, Lucas Pozzo-Miller.

This manuscript provides novel descriptions of enhanced dendritic spine formation and stabilization (especially in clusters) in L5 pyramidal neurons in primary motor cortex of MECP2 duplication mice under basal conditions and after rotarod motor learning. The manuscript provides evidence that in the MECP2 mouse model of autism new synaptic connections are more likely to be stabilized than in wild-type littermates and to appear on the dendritic shaft in closer proximity to each other. This abnormal behavior of dendritic spines may impair the ability of synaptic connections to adapt to new inputs and to properly store information, phenotypes commonly seen in autism spectrum disorder

The study entitled “Excessive synaptic clustering in the MECP2 duplication syndrome mouse model of autism” is a comprehensive analysis of the dynamics and structural clustering of newly formed dendritic spines in the MECP2 duplication syndrome mouse, a mouse model of autism spectrum disorder. The authors identify a significant increase in the formation and stabilization of dendritic spines and that these stabilized spines seem to appear closer to other new spines in the MECP2-duplication animals compared to wildtypes. This abnormal behavior of dendritic spines may impair the ability of synaptic connections to adapt to new inputs and to properly store information, phenotypes commonly seen in autism spectrum disorder. The authors observe improved motor learning in the mutant mice that correlates with increased stabilization of spines across genotypes. The approach and the analyses are straightforward. While the functional consequences of those structural differences are not addressed, the authors acknowledge the limitations of their study without further speculation. The manuscript is very well written, and the data are of sufficient quality to support the authors’ interpretations.  

Major comments:

 A piece of information missing is the quantification of transient spines, i.e., new spines formed observed at D5 that are no longer present at D9. Data in Fig. 1g (right panels) only address elimination of baseline spines, that to my understanding are those present at D1. If more spines are stabilized in the MECP2 mice, that means that less new spines are eliminated between D5 and D9, and the opposite for WT. That may balance the overall gain vs. loss metrics and therefore the spine density should not change significantly.

 Dovetailing with the previous comment, it seems that the elevated rate of spine formation together with a higher, although not significant, increase in spine elimination may render an overall increased turnover of dendritic spines. It would be informative for the readers to have that information available. It may be possible that an increase in the stabilization of newly formed spines coexists with an increase in turnover, in a way that favors elimination of previously existing spines. Jian et al., 2013 reported an increase in turnover in the same mouse model, how do the results from the current study align with them?

 It is a pity that the training did not induce any changes in spinogenesis or spine stabilization. Did, however, clustering of stabilized spines differ between trained and untrained animals?  It is not clear in the text, does the spines formed analysis include spines formed only at d5? What about d9?

Have the authors considered a way to confirm that the imaged spines have presynaptic partners?   Have the authors considered performing a quantitative analysis of spine morphology, perhaps spine volume?   What was the statistical Power yielded by the sample numbers used in all the experiments (post-hoc Power analysis)? This is important because some comparisons are described as “underpowered”. Also, the authors should explicitly state if investigators used criteria for data inclusion and exclusion (e.g.outliers), and if mice were randomly assigned to rotarod training.  

Minor comments:

 The 9 m cutoff for the cluster distance threshold sounds arbitrary. Why 9 and not 12 m? How did the authors determine when the data plateaued? A better justification for the 9 m cutoff is recommended.

 For the sake of transparency authors should explain the reason or reasons why figure 1i includes only 10 mice per group and why figure 1h includes additional mice. Ideally, figures 1e-g should show the individual data points and the data points presented in 1i somehow highlighted in panels e and (hollow vs. filled points). Same applies to figs 3f-g and the individual data points in figs 3c-e.

 The way the title currently reads may suggest that the excessive clustering of dendritic spines applies to all the spines, regardless of their persistence. A more specific title is recommended.

 ImageJ is not a MicroBrightField’s product

"Less than” instead of “less then” (P 6, p 2). Please correct

Scale bar missing in Fig 1c

 The authors need to state their rationale for using only male mice, even if the reason appears obvious.   The authors need to explain the meaning of “cooperative synaptic clustering”. What is it meant by “cooperative”?   The authors should not describe dendritic spines as “synapses” without a confirmation of either an adjacent presynaptic terminal or an immunolabled PSD on their heads.   The authors should include a description of the consequences of Mecp2 deletion on dendritic spine dynamics imaged in vivo (Landi et al. 2011), due to its close relationship to MECP2 overexpression.   The term “cytological” is more appropriate than “anatomical” when describing cellular features as dendritic spines (instead of features of a tissue).   All bar graphs should include individual data points.   Panels e and f in figure 1 lack the asterisk labels for statistical significance (as shown in panel g). They are also missing in figure 4.   Several citations are incomplete, lacking the journal volume and pages.

Note on the statistical analysis:

The authors mention that they were “underpowered” to detect statistical differences in a couple of comparisons, but do not provide information of how statistical Power was calculated, what was the Power threshold and why was it chosen, and measures can be taken to interpret the results of those comparisons.

## Author Response

Dear Dr. Hansel,

We are glad that our work was well-received by the reviewers, and we were highly motivated to address their issues and suggestions. The reviewers make several constructive and addressable issues with data analysis, statistics and presentation. We are sincerely appreciative of these comments, and in addressing them we feel that we have made the manuscript a more rigorous and readable scientific work.

In addition, we were approached by Christophe Bernard, the Editor-in-Chief, suggesting that we present our data using estimation statistics, a more rigorous way to visualize variability and statistical robustness in variable data sets (as is common in neuroscience). We explored the option and found it to be an exciting advance in data visualization. We therefore converted our primary data figures to be visualized as Gardner-Altman and Cummings estimation plots, following Ho et al., Nature Methods 2019; Bernard eNeuro 2019; and Calin-Jageman and Cumming eNeuro 2019. In the new Fig. 3c-e, data are presented as a Cumming estimation plot, with raw data plotted on the upper axes, and bootstrap-estimated distribution and 95% CI of the mean difference between groups plotted on the lower axes. In the new Fig. 4 and Extended Data Fig. 1-1, the left axis of each panel showed individual data points, and the right axis shows the bootstrapped distribution and 95% CI of the estimated difference between the groups. The old versions of Fig. 3 panels c-e are included as supplemental figures for completeness.

Here we reproduce the reviewers’ comments and our revisions point-by-point:

Major Comments A piece of information missing is the quantification of transient spines, i.e., new spines formed observed at D5 that are no longer present at D9. Data in Fig. 1g (right panels) only address elimination of baseline spines, that to my understanding are those present at D1. If more spines are stabilized in the MECP2 mice, that means that less new spines are eliminated between D5 and D9, and the opposite for WT. That may balance the overall gain vs. loss metrics and therefore the spine density should not change significantly. Dovetailing with the previous comment, it seems that the elevated rate of spine formation together with a higher, although not significant, increase in spine elimination may render an overall increased turnover of dendritic spines. It would be informative for the readers to have that information available. It may be possible that an increase in the stabilization of newly formed spines coexists with an increase in turnover, in a way that favors elimination of previously existing spines. Jiang et al., 2013 reported an increase in turnover in the same mouse model, how do the results from the current study align with them? We appreciate these suggestions very much. Indeed it is true that there is a decrease in elimination of new spines in mutant mice from D5 to D9, which parallels the increase in new spine stability. We did not show the newly-formed spine elimination directly because it is mathematically equivalent to the newlyformed spine stabilization (i.e. spine stabilization = 1 - spine elimination), which we provide in Fig. 1f,h. To address the larger and important point that the reviewer brings up, we explicitly analyzed dendritic spine turnover rate to allow comparison to Jiang et al 2013 and other previous studies, and indeed we found a significant increase in spine turnover rate in mutants (see new Fig. 1k), similar to the previous study in somatosensory cortex. There are therefore two plasticity processes acting differently in mutants, increased clustered spine stabilization and an overall increase in spine turnover, which serve to balance each other out. We updated our reporting and discussion of these results considerably (see updated Results paragraph 4, updated Discussion subsection on Elevated Clustered Spine Stabilization

and Increased Spine Turnover, page number 15-16), which we believe significantly improves the interest and readability of the manuscript. Again we are thankful for these suggestions and believe that the manuscript is now significantly improved.

Did, however, clustering of stabilized spines differ between trained and untrained animals? It is not clear in the text, does the spines formed analysis include spines formed only at d5? What about d9? We apologize that this was not clear. Similar to what was observed for overall spine formation and stabilization, clustered spine formation and stabilization did not differ between trained and untrained animals. We added the following sentence to the Results section to make this fact more explicit: “Similar to what was observed for overall spine formation and stabilization, analyzing trained and untrained animals separately showed no effect of training on clustered spine formation and stabilization (Formation: ANOVA effect of training: P=0.72, effect of genotype: P = 0.0008, interaction: P=0.3; Stabilization: ANOVA effect of training: P 0.59, effect of genotype: P <0.00001, interaction: P=0.9).” Additionally, as suggested by the reviewer below, we changed the data points so that trained mice are depicted as squares and untrained mice are depicted as circles. In this way, the reader can evaluate the effect of training independently in each comparison. We trained animals on the first four days and measured formation of clustered spines during this period (i.e. D1 to D5); we then tracked these spines from D5 to D9 to see how many of these stabilized. That is, clustered spine formation was measured on the first 4 days of imaging so that 1) we could determine any effect of training (which was only performed in the first 4 days) on formation and stabilization, and 2) we could track the behavior of newly formed spines from D5 to D9, to determine whether they stabilized.

Have the authors considered a way to confirm that the imaged spines have presynaptic partners? This is a fascinating line of investigation, and we have looked into methods to analyze presynaptic and postsynaptic partners in vivo. Fluorescence-based techniques to perform this (e.g. mGRASP, Kim et al. Nature Methods 2012) are appearing and are worthy future lines of investigation but are beyond the scope of the current work. We do know from prior post-hoc electron microscopy studies that dendritic spines which persist for at least 4 days essentially always form a synapse with a presynaptic axon (Knott et al., Nat Neuro 2006). Therefore we expect that the vast majority of the stabilized spines form a synapse. To note this fact we have added a sentence to the first paragraph of the Results section: “The follow-up imaging time point was chosen in line with prior studies showing that the vast majority of newly formed dendritic spines which persist for at least four days form an electron-microscopy-verified synapse (Knott et al., 2006).”

Have the authors considered performing a quantitative analysis of spine morphology, perhaps spine volume? This is an important suggestion. In a prior study (Jiang et al., 2013), we analyzed dendritic spine volume, neck width, and neck length, as well as analyzing spine density by morphological spine subtype (mushroom spines, stubby spines, and thin spines). We found that the loss of spine density that occurred in older animals (40 weeks) was due to a selective loss of stubby and thin spines (Compare Jiang et al Fig. 3H and 4C). We also found subtle differences in spine morphology that was developmentally dependent (see Jiang et al Fig. 5), but no changes that were dramatic enough that we thought it essential to repeat these analyses in the current study.

What was the statistical Power yielded by the sample numbers used in all the experiments (post-hoc Power analysis)? This is important because some comparisons are described as “underpowered”. In the original manuscript, the lack of statistical power for the spine elimination rate was noted without reporting the actual statistical power. Therefore in the primary comparisons we now report the statistical power (1-β) based on the WT mean, SD, and sample size. Using the threshold power of 0.8 (equivalent to an alpha of 0.05), we find that the study was adequately powered to detect changes in spine formation (1-β =0.994) and stabilization (1-β =0.9991), but not spine elimination (1-β=0.60 for d1 to d5 and 0.64 for d5 to day 9). A sample size of ∼22 animals would therefore be needed to detect a significant difference in spine elimination, based on the estimated WT SD. The manuscript has been updated to include these considerations.

the authors should explicitly state if investigators used criteria for data inclusion and exclusion (e.g.outliers), and if mice were randomly assigned to rotarod training. A new section was added to the Methods section describing data inclusion criteria: “Data inclusion was determined by the following indicators of imaging quality: 1) low background noise across all time points, 2) <5 pixel [0.25 μm] motion artifact, and 3) dendrites well isolated from other fluorescent structures. Mice for which imaging quality significantly degraded during the time course of imaging were excluded from the analysis. Otherwise all data was included, and no statistical outliers were excluded.” Regarding assignment to rotarod vs rest condition: Mice were imaged in the training and rest conditions in cohorts by litter. All animals within a litter were imaged in either the training or rest condition blind to genotype. Imaging of trained mice generally occurred earlier in the overall course of experiments, and then subsequently imaging of untrained mice occurred as a necessary control condition. Other than training, experimental conditions were identical between the two groups.

Minor comments: The 9 um cutoff for the cluster distance threshold sounds arbitrary. Why 9 and not 12um? How did the authors determine when the data plateaued? A better justification for the 9 m cutoff is recommended. The difference in clustered spines stabilized between mutant and WT increased with increasing cluster distance threshold from 1 μm up to 9 μm before mostly leveling off; 10-12 μm could have also reasonably been chosen. Importantly, we analyzed the data for a range of cluster thresholds (5-to12μm) and found similar statistically significant difference for a range of values. We decided to settle on 9 microns because 1) it is where the overall trend of the difference plateaus; 2) is most similar to a known form of cooperative synaptic potentiation described by Harvey and Svoboda (Harvey et al., Nature 2007, Harvey et al., Science 2008), and 3) it is also in the range of training-induced synaptic clustering described in Fu et al. Nature 2012. Twelve μm was slightly outside the range of clustering described in these two previous studies. These facts lead us to believe that it is an appropriate, although slightly arbitrary, threshold for the report. To somewhat address the reviewer’s concern, we added the following statement to the Results section: “Importantly, similar statistically significant results were observable for a range of cluster thresholds (from 5 to 12μm).” We will defer to the Editors on whether or not additional changes are necessary here.

For the sake of transparency authors should explain the reason or reasons why figure 1i includes only 10 mice per group and why figure 1h includes additional mice.

Fig. 1i (now 1l in the updated manuscript) has more animals than the other panels (n=22 and 17 vs n=10 and 10), because mice excluded from the imaging analysis due to issues with imaging quality but who nevertheless underwent rotarod training were also included in this panel. We have made a note in the figure legend to explain this point as suggested.

figures 1e-g should show the individual data points and the data points presented in 1i somehow highlighted in panels e and (hollow vs. filled points). Same applies to figs 3f-g and the individual data points in figs 3c-e. Fig. 1 and 3 have been updated such that trained mouse data points are represented as squares and untrained mouse data points are represented as circles, so that readers can easily determine which mice were trained or not throughout the manuscript.

The way the title currently reads may suggest that the excessive clustering of dendritic spines applies to all the spines, regardless of their persistence. A more specific title is recommended. We closely considered this suggestion. In discussion with the authors, we decided to change the title of the manuscript to “Excessive formation and stabilization of dendritic spine clusters in the MECP2 duplication syndrome mouse model of autism”. We feel this new title more specifically describes the report’s major findings.

ImageJ is not a MicroBrightField’s product... “Less than” instead of “less then” (P 6, p 2). Please correct ... Scale bar missing in Fig 1c. Thank you for these suggested edits. They were implemented.

The authors need to state their rationale for using only male mice, even if the reason appears obvious. The Methods section has been modified to include the sentence “Males were used in experiments, to avoid possible changes in turnover related to estrous cycle in females (Alexander et al., 2018)", citing the paper that shows that sensory-evoked spine plasticity is sensitive to the animal’s estrous cycle.

The authors need to explain the meaning of “cooperative synaptic clustering”. What is it meant by “cooperative”? By cooperative we are referring to the concept of cooperativity, in which elements of a system act dependently on each other rather than independently. In this case, clusters of newly formed dendritic spines act cooperatively to favor each other’s stabilization, in mutants. We have examined the use of ‘cooperative’ in the manuscript, defined it in the introduction, and taken steps to ensure that this use of the term is more clearly established.

The authors should not describe dendritic spines as “synapses” without a confirmation of either an adjacent presynaptic terminal or an immunolabled PSD on their heads. As described above, we do know from prior post-hoc electron microscopy studies that dendritic spines which persist for at least 4 days always form a synapse with a presynaptic axon (Knott et al., Nat Neuro 2006). Therefore we expect that the vast majority of the stabilized spines form a synapse. To note this fact we have added a sentence to the first paragraph of the Results section: “The follow-up imaging time point was chosen in line with prior studies showing that the vast majority of newly formed dendritic

spines which persist for at least four days form an electron-microscopy-verified synapse (Knott et al., 2006).” Nonetheless, it is true that we cannot exclude the possibility that the MECP2 duplication mouse may behave differently from WT animals. We therefore updated the manuscript throughout to use the term ‘dendritic spine’ rather than ‘synapse’ where appropriate.

The term “cytological” is more appropriate than “anatomical” when describing cellular features as dendritic spines (instead of features of a tissue). This is a reasonable suggestion. The manuscript has been updated to use the cytological rather than anatomical throughout.

The authors should include a description of the consequences of Mecp2 deletion on dendritic spine dynamics imaged in vivo (Landi et al. 2011), due to its close relationship to MECP2 overexpression. A reference to this relevant paper was added to the Introduction.

All bar graphs should include individual data points. The manuscript has been updated to include individual data points in all figures.

Panels e and f in figure 1 lack the asterisk labels for statistical significance (as shown in panel g). They are also missing in figure 4. This suggestion has been implemented, much appreciated.

Several citations are incomplete, lacking the journal volume and pages. We are grateful that the reviewer checked the bibliography since we started using a different reference manager. We believe that the references are now complete.

The authors mention that they were “underpowered” to detect statistical differences in a couple of comparisons, but do not provide information of how statistical Power was calculated, what was the Power threshold and why was it chosen, and measures can be taken to interpret the results of those comparisons. As above: In the original manuscript, the lack of statistical power for the spine elimination rate was noted without reporting the actual statistical power. Therefore in the primary comparisons we now report the statistical power (1-β) based on the WT mean, SD, and sample size. Using the threshold power of 0.8 (equivalent to an alpha of 0.05), we find that the study was adequately powered to detect changes in spine formation (1-β =0.994) and stabilization (1-β =0.9991), but not spine elimination (1β=0.60 for d1 to d5 and 0.64 for d5 to day 9). A sample size of ∼22 animals would therefore be needed to detect a significant difference in spine elimination, based on the estimated WT SD. The manuscript has been updated to include these considerations.
