# Supplementary figures and images for: Excessive Formation and Stabilization of Dendritic Spine Clusters in the MECP2-Duplication Syndrome Mouse Model of Autism
Source: eNeuro. 2021 Jan 28;8(1):ENEURO.0282-20.2020. doi: 10.1523/ENEURO.0282-20.2020 (PMC7877475; doi:10.1523/ENEURO.0282-20.2020)

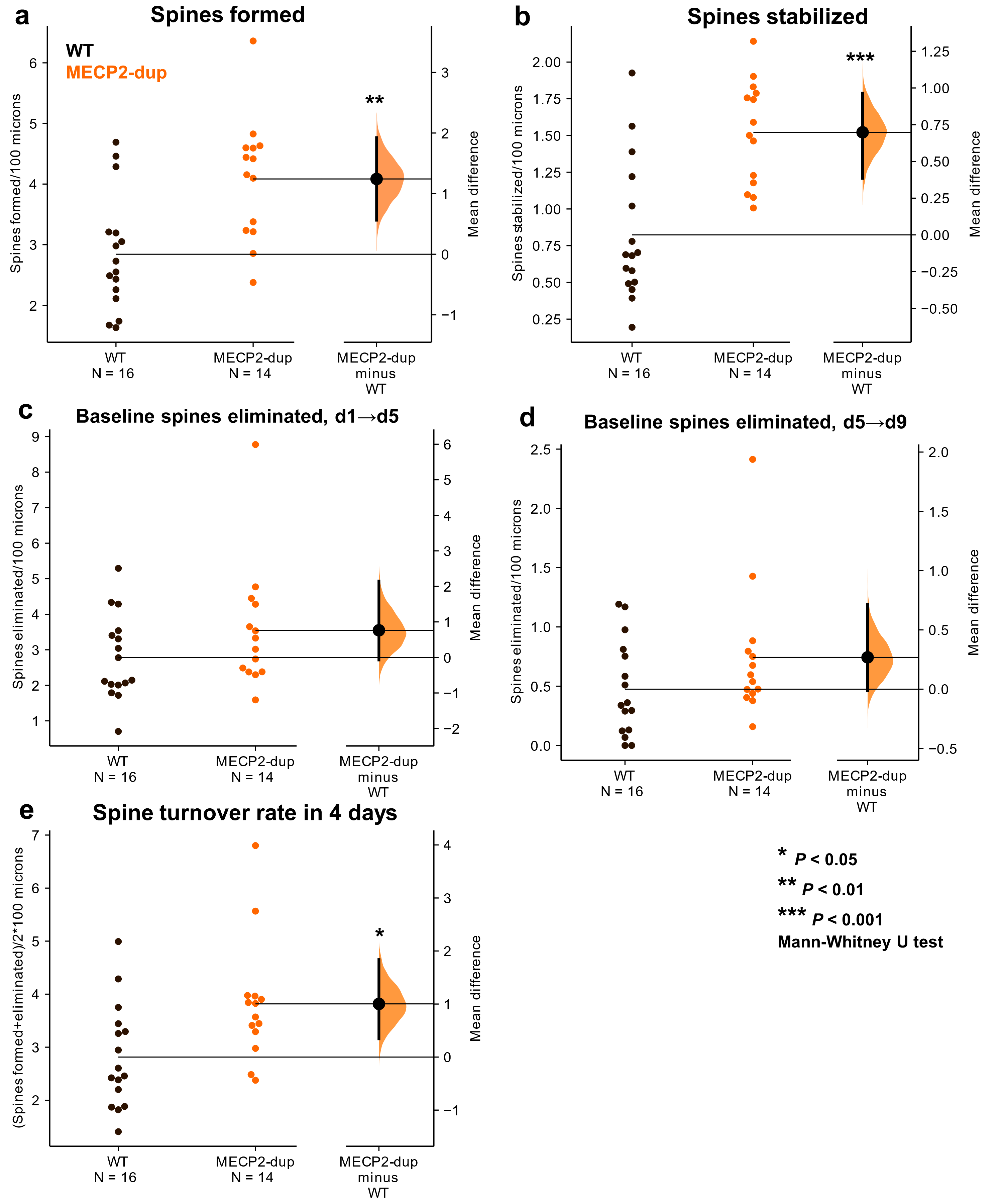

Supplement: Extended Data Figure 1-1 — Dendritic spine structural plasticity estimation statistics. A–E, Same as Figure 1G–K, but plotted as a Gardner–Altman estimation plot to visualize the results using estimation statistics as in Ho et al. (2019). The left axis of each panel shows individual data points for WT (black) and MECP2-duplication (orange) animals. The right axis shows the bootstrapped distribution (light orange) and 95% confidence interval (vertical black line) of the estimated difference between the two groups; *p < 0.05, **p < 0.01, ***p < 0.001, Mann–Whitney U test. Download Figure 1-1, TIF file. [file enu-eN-NWR-0282-20-s01.tif]

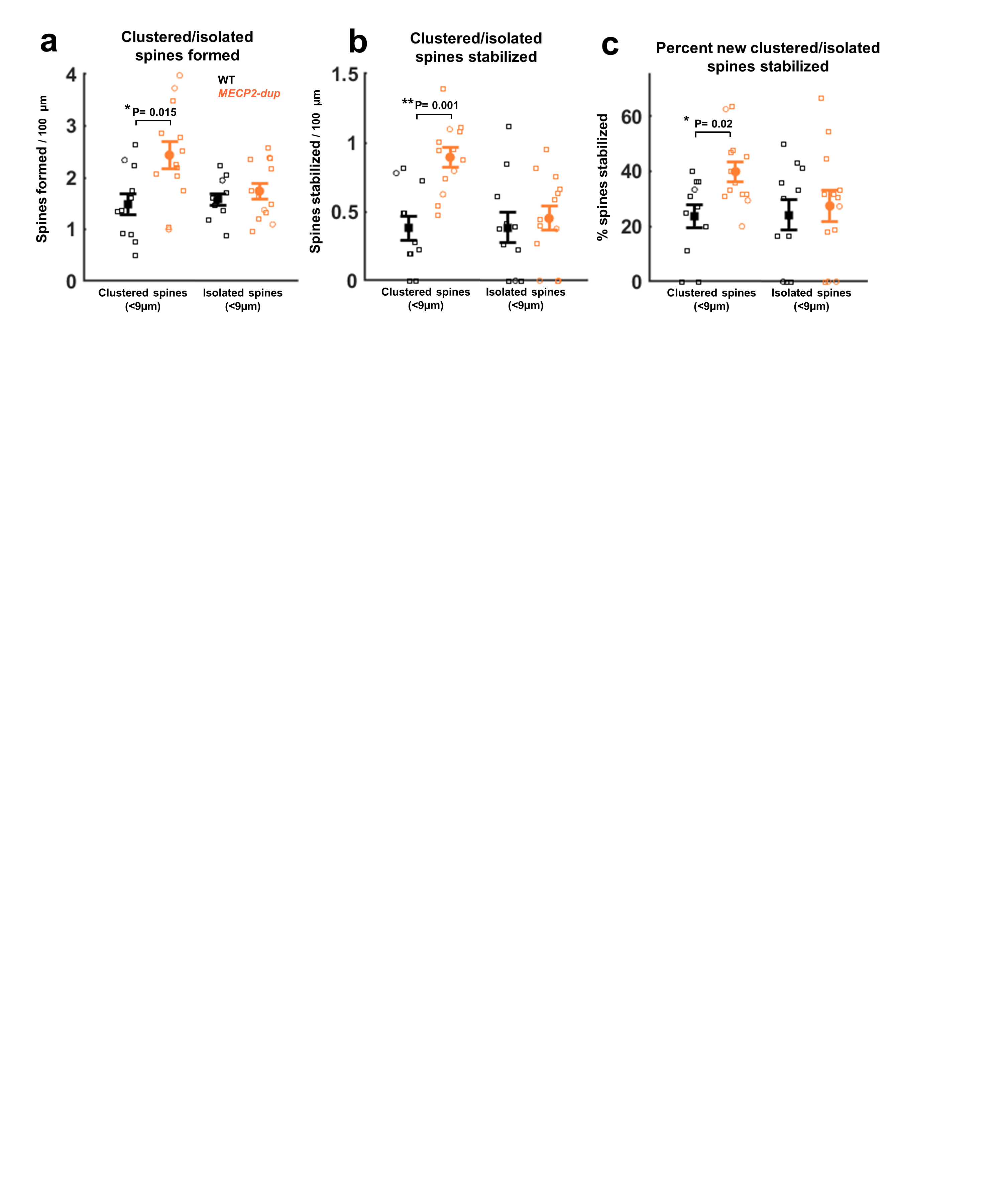

Supplement: Extended Data Figure 3-1 — Clustered and isolated spine stabilization supplemental plots. A–C, Same as Figure 3C–E, but plotted as mean ± error bars, with individual animal data points plotted as squares (trained) and circles (untrained). Download Figure 3-1, TIF file. [file enu-eN-NWR-0282-20-s02.tif]
